# Coupling of glucose metabolism with mitophagy via O-GlcNAcylation of PINK1

**DOI:** 10.7150/ijbs.112672

**Published:** 2025-06-20

**Authors:** Zhiwei Xu, Xiangzheng Gao, Dade Rong, Jingyao Wang, Liangliang Gao, Mingzhu Tang, Yiguan Chen, Yichi Zhang, Liming Xie, Liming Wang, Guang Lu, Jia-Hong Lu, Wei Liu, Han-Ming Shen

**Affiliations:** 1 Faculty of Health Sciences, Ministry of Education Frontiers Science Center for Precision Oncology, University of Macau, Macau, China.; 2 Jiangsu Key Laboratory of Medical Science and Laboratory Medicine, Department of Laboratory Medicine, School of Medicine, Jiangsu University, Zhenjiang, China.; 3 School of Biomedical Sciences, Hunan University, Changsha, China.; 4 Department of Physiology, Zhongshan School of Medicine, Sun Yat-sen University, Guangzhou, China.; 5 State Key Laboratory of Quality Research in Chinese Medicine, Institute of Chinese Medical Sciences, University of Macau, Macau, China.; 6 Center for Metabolism Research, The Fourth Affiliated Hospital of Zhejiang University School of Medicine, and International School of Medicine, International Institutes of Medicine, Zhejiang University, Yiwu, China.

**Keywords:** glucose metabolism, HBP, mitophagy, O-GlcNAcylation, PINK1

## Abstract

Mitophagy is a selective form of autophagy for the clearance of damaged and dysfunctional mitochondria via the autophagy-lysosome pathway. As mitochondria are the most important metabolic organelles, the process of mitophagy is tightly regulated by glucose metabolism. At present, it is known that glucose is required for the mitophagy process, while the underlying mechanisms remain to be further elucidated. In this study, we establish a novel regulatory role of glucose metabolism in mitophagy via protein O-GlcNAcylation. First, we found that acute mitochondrial damage enhanced glucose uptake and promoted protein O-GlcNAcylation. Second, we provided evidence that protein O-GlcNAcylation promotes PINK1-Parkin-dependent mitophagy. Next, we attempted to illustrate the molecular mechanisms underlying the regulation of O-GlcNAcylation in mitophagy by focusing on PTEN-induced kinase 1 (PINK1). One important observation is that PINK1 is O-GlcNAcylated upon acute mitochondrial damage, and suppression of O-GlcNAcylation impairs PINK1 protein stability and its phosphorylated ubiquitin, leading to impaired mitophagy. More importantly, we found that glucose metabolism promotes mitophagy via regulating O-GlcNAcylation. Taken together, this study demonstrates a novel regulatory mechanism connecting glucose metabolism with mitophagy via O-GlcNAcylation of PINK1. Therefore, targeting the O-GlcNAcylation may provide new strategies for the modulation of mitophagy and mitophagy-related human diseases.

## Introduction

Autophagy, or macroautophagy, refers to an evolutionarily conserved process in which autophagosome engulfs various cytosolic components for lysosome-dependent degradation 1, 2. Mitophagy is a selective form of autophagy for the clearance of damaged or dysfunctional mitochondria via the autophagy-lysosome pathway 3, 4. Owing to its critical role in mitochondrial homeostasis and quality control, impaired mitophagy has been closely implicated in the pathological development of various diseases, including neurodegenerative disorders and cancer 5.

One of the best-characterized mitophagy machinery is the ubiquitin (Ub)-dependent mitophagy via PINK1 (PTEN-induced kinase 1)-Parkin pathway 4, 6. PINK1 is a mitochondrial kinase and Parkin is an E3 ligase 6, 7. Under normal conditions, PINK1 is constitutively kept at a low level via cleavage and proteasomal degradation. Under mitochondrial stress, PINK1 is stabilized and activated at the outer membrane of the damaged mitochondria where it phosphorylates Ub and Parkin, which results in the mitochondrial recruitment and activation of Parkin 8-10. PINK1 and Parkin then constitute a positive feedback loop, resulting in generation of Ub chains on the outer mitochondrial membrane (OMM) 11, 12 and the subsequent recruitment of cargo receptors such as calcium-binding and coiled-coil domain 2 (CALCOCO2/NDP52) and coiled-coil containing protein optineurin (OPTN), which facilitates the engulfment of damaged mitochondria into autophagosome and their ultimate degradation in the lysosome 6, 13, 14. Based on the current model, phosphorylation and ubiquitination mediated by PINK1 and Parkin, respectively, are the two most important forms of post-translational modifications (PTMs) in mitophagy, while the implication of other forms of PTMs, such as glycosylation in mitophagy is much less studied.

Protein glycosylation refers to a process by which a carbohydrate is covalently attached to a target protein 15. Among many types of glycosylation, O-GlcNAcylation appears to be particularly important in the regulation of cellular functions 16, 17. O-GlcNAcylation is mediated by two key enzymes: OGT (O-GlcNAc transferase) and OGA (O-GlcNAcase), controlling the attachment or removal of the O-GlcNAc moiety to the serine and threonine hydroxyl groups, respectively 18-20. At present, O-GlcNAcylation has been shown to play an important role in mitochondrial homeostasis 21-23. For instance, enhanced cellular O-GlcNAcylation in OGA-knockout cells led to significant changes in mitochondrial morphology, mitochondrial fission, and ETC complex activities 24. O-GlcNAcylation of mitochondrial proteins, including dynamin-related protein 1 (DRP1) and fusion-related mitochondrial protein OPA1, has been linked to mitochondrial fragmentation 25, 26. Importantly, there is evidence linking O-GlcNAcylation with mitophagy, based on the observation that defective O-GlcNAcylation in OGT-deficient hematopoietic stem and progenitor cells impairs mitophagy due to downregulation of PINK1 27. Intriguingly, OGT and O-GlcNAcylation have also been reported to play a negative role in mitophagy, based on the observations that OGT inhibition led to the increased level of mitophagy evidenced by the higher level of mitochondrial protein degradation 28. Therefore, more work is needed to explore the exact role of O-GlcNAcylation in mitophagy.

On the other hand, glucose metabolism is known to play a regulatory role in mitophagy. For instance, glucose starvation inhibits mitophagy via the suppression of PINK1 translation 29. Moreover, some key enzymes in glycolysis have been implicated in mitophagy. Hexokinase 2 (HK2) which converts glucose into glucose 6-phosphate (Glc-6P) has been shown to mediate PINK1 complex assembly and activation 30. More recently, another glycolytic enzyme, PKM2, promotes mitophagy by facilitating mitochondrial fission via the AMPK-mTOR pathway 31. Notably, the hexosamine biosynthetic pathway (HBP) is a glucose metabolism pathway that results in the synthesis of a nucleotide sugar uridine diphosphate-N-acetyl glucosamine (UDP-GlcNAc), which is subsequently used for O-GlcNAcylation 32. To date, it remains to be studied whether O-GlcNAcylation contributes to the regulatory roles of glucose metabolism in mitophagy.

Here, we report a novel mechanism by which glucose metabolism promotes mitophagy via O-GlcNAcylation: upon mitochondrial damage, PINK1 undergoes O-GlcNAcylation via direct interaction with OGT, which facilitates the stabilization and activation of PINK1. Our study thus establishes a new link between glucose metabolism and mitophagy and suggests the potential of targeting O-GlcNAcylation in human diseases associated with dysregulation of PINK1-dependent mitophagy.

## Results

### Acute mitochondrial damage enhances glucose uptake and promotes protein O-GlcNAcylation

**Figure 1:**
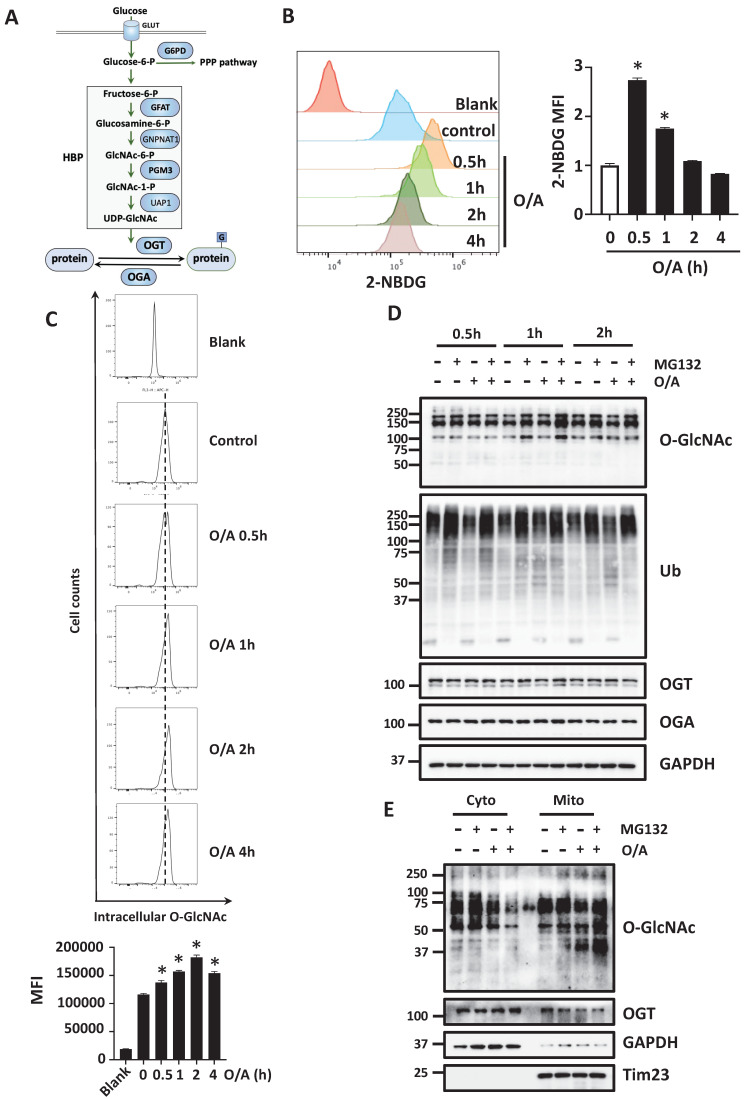
** Increased glucose uptake and protein O-GlcNAcylation in YFP-HeLa cells.** (**A**) Flow cytometry assay of glucose uptake by YFP-HeLa cells in the 2-NBDG assay. (**B**) with statistical data showing signals in the left panel. MFI, mean fluorescence intensity. (**C**) Flow cytometric analysis of intracellular O-GlcNAc levels in YFP-HeLa treated with O/A (1μM and 1μM) for 0.5 to 4 h. (**D**) YFP-Parkin expressing HeLa cells were pretreated with or without MG132 (10 μM) for 1 h. Subsequently, the cells were treated with or without O/A (1μM and 1μM) for 0.5, 1, 2 h and subjected to western blotting analysis with the indicated antibodies. (**E**) YFP-HeLa cells were pretreated with or without MG132 (10 μM) for 1 h, then treated with or without 1 μM O/A for 1 h. Subcellular fractionation was then performed to isolate the cytosolic and mitochondrial fractions. Tim23 and GAPDH were used as mitochondrial and cytosolic markers, respectively. *p < 0.05.

Glucose is known to be required for mitophagy, based on the fact that Parkin-dependent mitophagy induced by mitochondrial damage is completely blocked upon glucose deprivation 29. Glucose is also the major source that produces protein O-GlcNAcylation via HBP 18, 33. Sequential reactions are catalyzed by several metabolic enzymes to produce cellular UDP-GlcNAc. Protein O-GlcNAcylation is only cycled on and off proteins via a single pair of OGT and OGA, respectively (Figure 1A). To understand the potential role of glucose metabolism in mitophagy, we first tested the effects of mitochondrial damage on cellular glucose uptake. To do this, we treated HeLa cells stably expressing YFP-Parkin (hereafter referred to as YFP-HeLa) with mitochondrial damaging agents oligomycin and antimycin A (O/A), and then performed a glucose uptake assay using a glucose analog 2-(N-(7-nitrobenz-2-oxa-1,3-diazol-4-yl) amino)-2-deoxyglucose (2-NBDG). We first found an initial increase of glucose uptake following O/A treatment (Figure 1B). Similar results were found in wild-type (WT) HeLa cells without Parkin expression (Figure S1A-1B). When mitochondria are damaged, mitochondrial proteins quickly undergo degradation via proteasome and lysosome. Correlated roughly with the glucose uptake, the levels of cellular O-GlcNAcylation were increased when mitochondrial depolarization was induced by O/A and carbonyl cyanide chlorophenylhydrazone (CCCP) (Figure 1C and S1C). Next, we analyzed the global O-GlcNAcylation in YFP-HeLa cells upon treatment with O/A together with proteasome inhibitor MG132, and found an increased level of O-GlcNAcylation (Figure 1D). A similar result was found when the cells were treated with O/A together with an autophagy-lysosome inhibitor bafilomycin (Figure S1D). Intriguingly, the increased O-GlcNAcylation was only found in the mitochondrial proteins, in comparison to the cytosolic proteins (Figure 1E). Consistently, following the topology assay of the mitochondrial fraction from YFP-HeLa cells, we found that OGT mainly localizes at the OMM, similar to Tom20 and Tom40 (Figure S1E). These results thus suggest that upon mitochondrial damage, cells undergo increased glucose metabolism and protein O-GlcNAcylation.

### Suppression of O-GlcNAcylation impairs mitophagy

**Figure 2:**
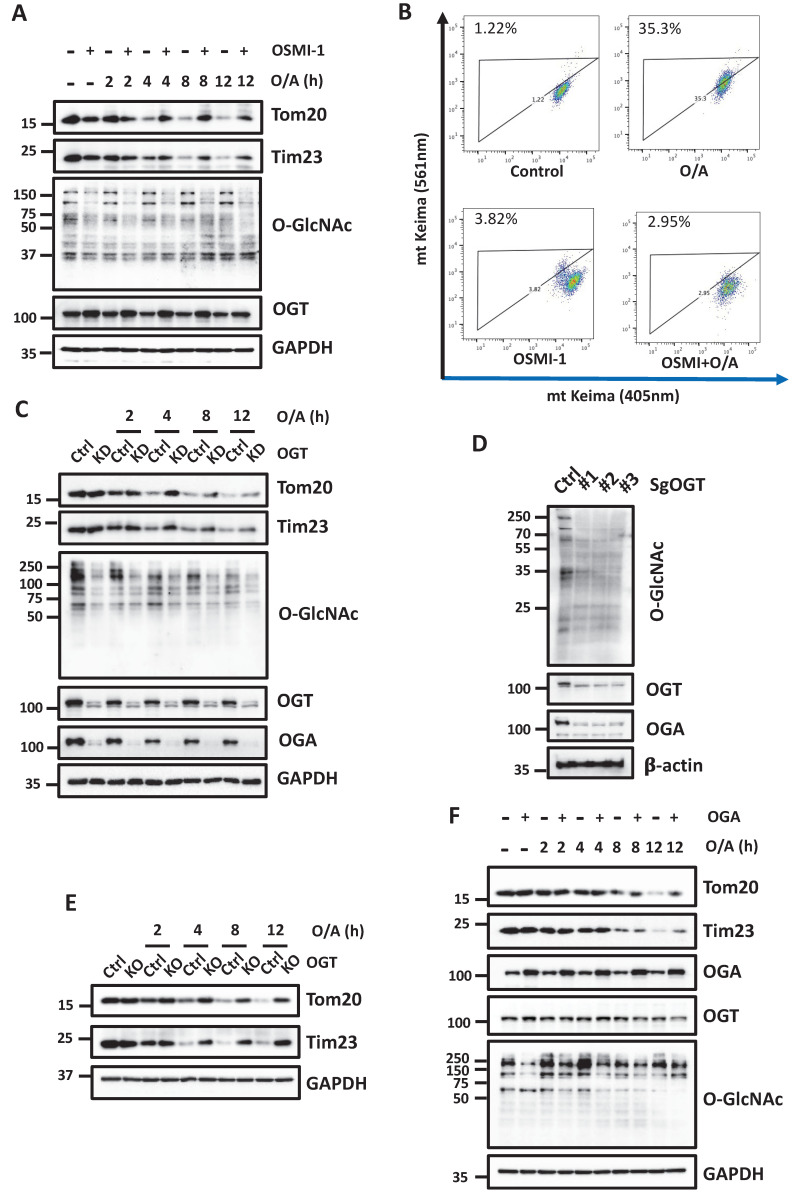
** Disruption of O-GlcNAcylation inhibits O/A-induced mitophagy.** (**A**) YFP-HeLa cells were pretreated with OSMI-1 (50 μM) for 12 h, then treated with 1 μM O/A and harvested at the indicated time points for western blotting analysis with the indicated antibodies. (**B**) Cells were pretreated with or without OSMI-1 for 24 h, and treated with 1 μM O/A for 4 h, then analyzed by FACS for lysosomal positive mt-mKeima. (**C**) YFP-HeLa cells or OGT knockdown cells were treated with 1 μM O/A for the indicated time, were then lysed for western blotting analysis. (**D**) Mcherry-Parkin expressing control HeLa cells or OGT knockout cells were lysed for western blotting analysis to confirm the knockout effects of OGT. (**E**) Mitochondria content was determined by immunoblotting of proteins from OMM and IMM compartments in control and OGT KO cells. (**F**) YFP-HeLa cells were overexpressed with or without OGA, and were treated with 1 μM O/A and harvested at the indicated time points for immunoblotting. (**G**) Mcherry-Parkin expressing HeLa or GFAT1-KO cells were treated with 1 μM O/A and harvested at the indicated time points for western blotting analysis.

To further understand the potential regulatory role of O-GlcNAcylation in mitophagy, we modulated the O-GlcNAcylation level using both pharmacological and genetic approaches targeting OGT. Upon mitophagy induction, a pharmacological inhibitor of OGT, OSMI-1, significantly attenuated the degradation of the mitochondrial proteins such as Tom20 and Tim23 (Figure 2A). Alternatively, OSMI-1 suppressed mitophagy induced by O/A in YFP-HeLa cells, measured by mt-Keima assay, a well-established quantitative method to measure mitophagy (Figure 2B). Next, we used genetic approaches to knockdown (KD) OGT. As shown in Figure 2C, OGT KD significantly reduced the O-GlcNAcylation level and attenuated the degradation of OMM and IMM proteins following O/A treatment. In cells with OGT knockdown, OGA protein levels are significantly downregulated 34, 35. Consistently, OGA was dramatically reduced in YFP-HeLa cells knocking down or knocking out OGT compared with the control cells (Figure 2C- 2D). To further establish the importance of OGT in PINK1-Parkin-mediated mitophagy, we generated OGT KO cells using the CRISPR-Cas9 technique. Three different sgRNA sequences targeting OGT were chosen to confirm the knockout effects on OGT and O-GlcNAcylation level (Figure 2D). Consequently, OGT KO significantly attenuated the reduction of Tom20 and Tim23 upon O/A treatment (Figure 2E). Since OGA catalyzes the removal of this sugar modification, here we further manipulated the O-GlcNAcylation level by OGA overexpression. HeLa cells overexpressing OGA showed similar phenotypes as OGT KD or KO: lower levels of O-GlcNAcylation and impaired mitochondrial protein degradation (Figure 2F). However, mitochondrial proteins are degraded in a similar manner in OGA KO cells compared with the WT (Figure S2). Thus, data from this part of our study clearly demonstrated that O-GlcNAcylation positively regulates mitophagy.

### O-GlcNAcylation contributes to PINK1 stabilization and activation at mitochondria

**Figure 3:**
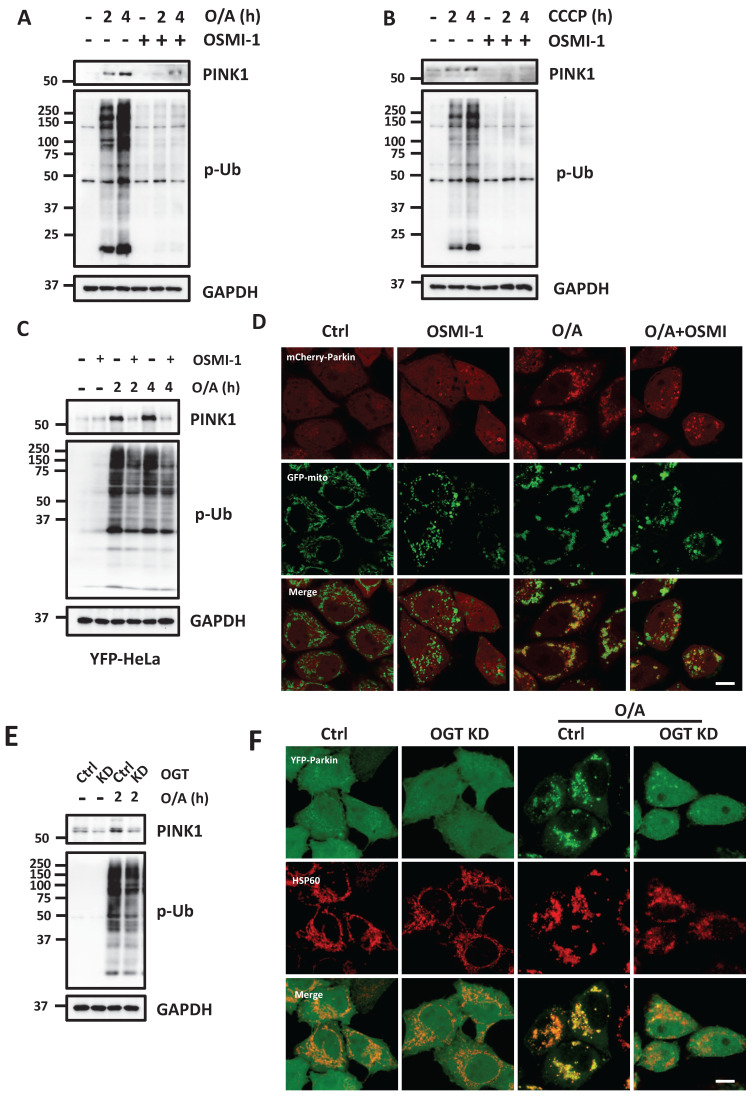
** O-GlcNAcylation promotes PINK1 level and its phosphorylated ubiquitin.** (**A and B**) HEK293T cells were pretreated with OSMI-1 (50 μM) for 24h, then were treated with 1 μM O/A or 20 μM CCCP and harvested at the indicated time points for western blotting analysis with the indicated antibodies. (**C**) YFP-HeLa cells were pretreated with OSMI-1 (50 μM) for 24 h, then were treated with 1 μM O/A and harvested at the indicated time points for western blotting analysis. (**D**)Representative images of mCherry-Parkin, GFP-mitochondrial expressing HeLa cells pretreated with OSMI-1 for 24h and treated with O/A for 2 h. (**E**) YFP-HeLa or OGT KD cells were treated with 1 μM O/A and harvested at 2 h for western blotting analysis. (**F**) Representative images of YFP-HeLa and OGT KD cells treated with O/A for 2h as indicated by YFP-Parkin and mitochondrial marker HSP60. Scale bar=10μm.

To understand the molecular mechanisms underlying the regulatory role of O-GlcNAcylation in mitophagy, we analyzed the impact of O-GlcNAcylation on PINK1 and Parkin, two key regulators of mitophagy. Upon O/A treatment of HEK293T cells, full-length PINK1 stabilizes at the OMM and phosphorylates Ub, while the presence of an OGT inhibitor, OSMI-1, markedly reduced both full-length PINK1 and p-Ub levels (Figure 3A-3B). Similar results were found in YFP-HeLa cells with exogenous Parkin (Figure 3C). Consistently, Parkin recruitment to mitochondria was also significantly impaired in the presence of OSMI-1 (Figure 3D and S3A). To further validate the effect of O-GlcNAcylation on PINK1, we next used OGT-KD cells, and as expected, OGT KD significantly reduced full-length PINK1 and p-Ub following O/A treatment (Figure 3E). Consistently, Parkin recruitment to mitochondria was also suppressed in the OGT-KD and OGT KO cells in response to O/A treatment (Figure 3F, S3B-D). Since PINK1 stabilization is directly caused by impairment of MMP, we constructed the OGT KD in HEK293T cells (Figure S3E) and measured MMP, and found that OGT KD had no evident effect on MMP with or without O/A treatment (Figure S3F). Hence, these data indicate that O-GlcNAcylation promotes mitophagy via stabilization and activation of PINK1 independent of MMP.

### Inhibition of HBP impedes mitophagy

**Figure 4:**
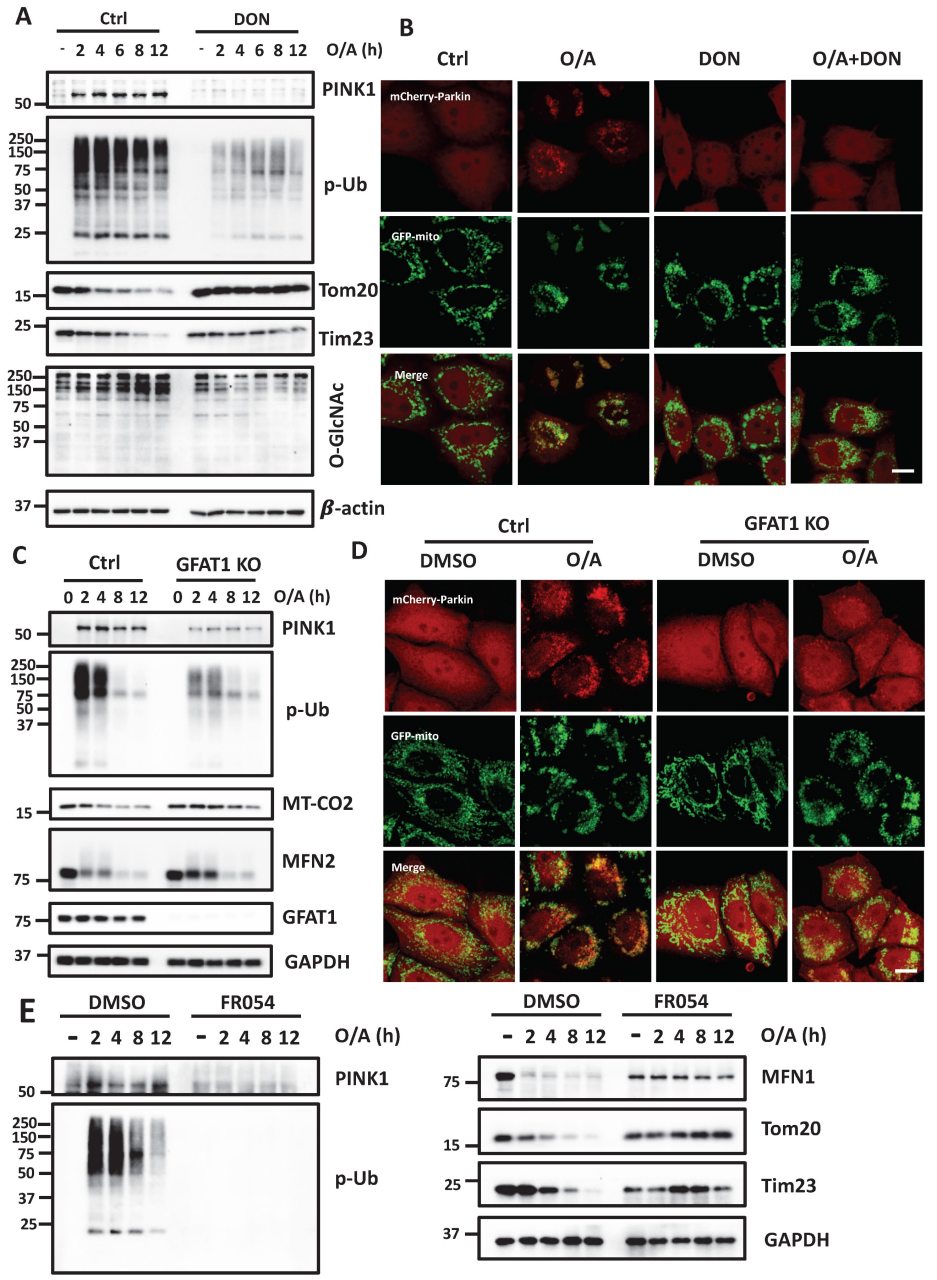
** Mitophagy is impaired when HBP enzyme is inhibited.** (**A**) YFP-HeLa cells were pretreated with DON (100 μM) for 12 h, then treated with 1 μM O/A and harvested at the indicated time points for western blotting analysis with the indicated antibodies. (**B**) Representative images of mCherry-Parkin, GFP-mitochondrial expressing HeLa cells pretreated with DON (100 μM) for 24h and treated with O/A for 2 h. Scale bar=10μm. (**C**) PINK1, P-Ub, and mitochondrial membrane proteins were determined by immunoblotting in control and GFAT1-KO cells upon O/A induction. (**D**) mcherry-Parkin, GFP-mitochondrial expressing HeLa cells treated with 1 μM O/A for 2 h compared with GFAT1-KO cells, and representative images were taken. Scale bar=10μm. (**E**) YFP-HeLa cells were pretreated with FR054 (1mM) for 12 h, then treated with 1 μM O/A and harvested at the indicated time points for western blotting analysis with the indicated antibodies.

Having confirmed the positive contributory role of O-GlcNAcylation in mitophagy, we next attempted to examine the upstream regulators of OGT and OGA in the HBP pathway. Glutamine-fructose-6-phosphate amidotransferase 1 (GFAT1) is the first rate-limiting enzyme in the HBP pathway, which catalyzes the substrate fructose-6-phosphate (Fru-6P) to yield glucosamine-6-phosphate (GlcN-6P) 36. We tested the effect of a GFAT1 inhibitor, DON, and confirmed that DON greatly reduced protein O-GlcNAcylation. Upon mitophagy induction by O/A, GFAT1 inhibition significantly attenuated PINK1 accumulation and the following upregulation of p-Ub, thereby impairing the degradation of the mitochondrial proteins (Figure 4A). Inhibition of GFAT1 following DON treatment prevented Parkin translocation to damaged mitochondria in response to O/A (Figure 4B and S4A). We next tested the possible role of GFAT1 in mitophagy by establishing GFAT1 KO cells. Deletion of GFAT1 markedly reduced O-GlcNAcylation and mitochondrial protein degradation in cells treated with O/A (Figure 4C), indicating that O-GlcNAcylation mediated by GFAT1 is required for mitophagy. Consistently, Parkin translocation to mitochondria was suppressed in GFAT1 KO cells (Figure 4D and S4B). Phosphoglucomutase 3 (PGM3) is the key enzyme that produces GlcNAc-1-P in the third step 37. Similarly, defects in mitophagy occur when PGM3 was inhibited by a chemical inhibitor, FR054, to suppress O-GlcNAcylation (Figure 4E).

### PINK1 is O-GlcNAcylated by OGT during mitophagy

**Figure 5:**
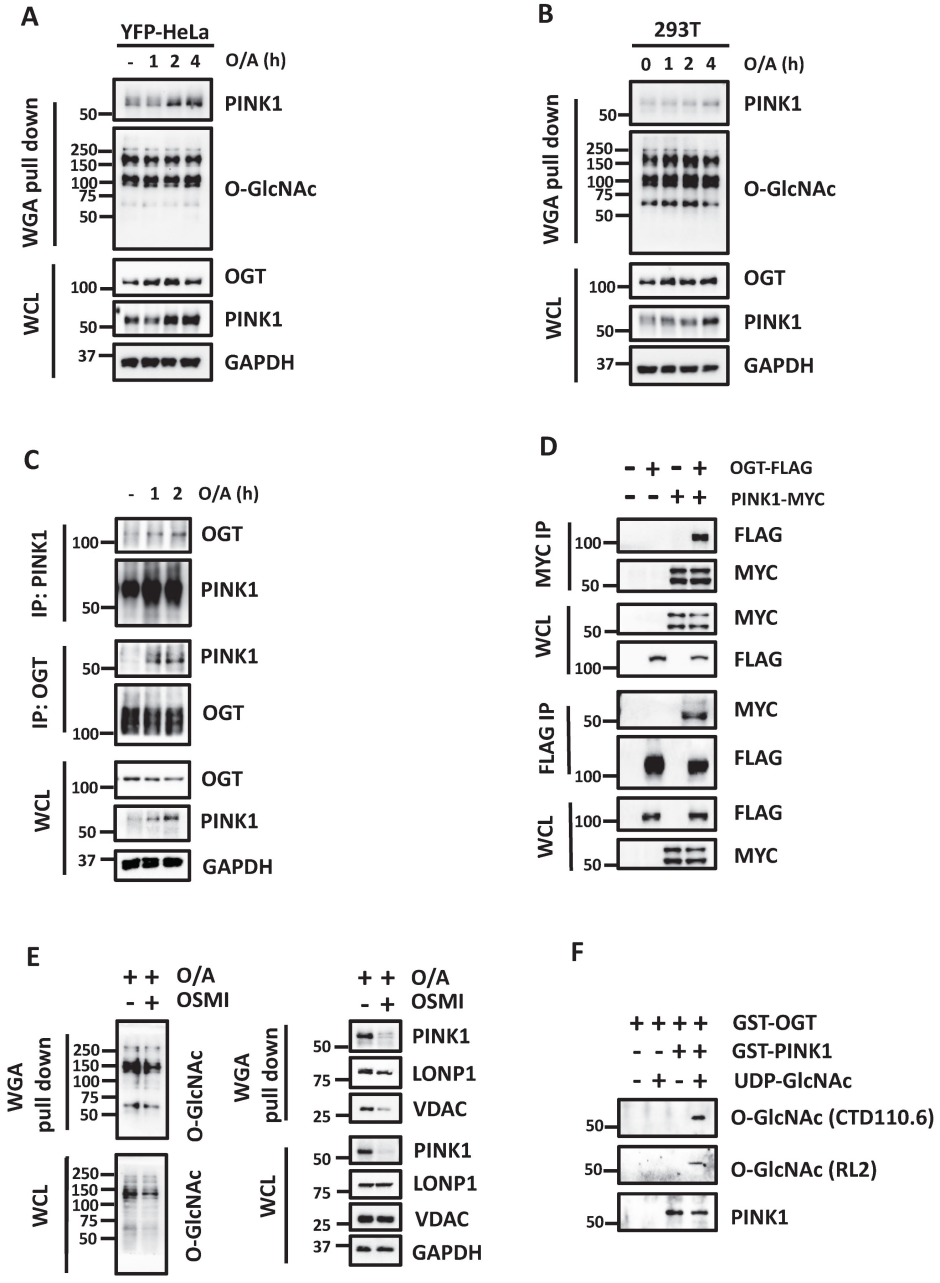
** PINK1 protein is O-GlcNAcylated and interacts with OGT.** (**A and B**) YFP-HeLa or HEK293T cells were treated with 1 μM O/A for the indicated time points. WGA was used to affinity pull-down O-GlcNAcylated proteins, and then immunoblot to detect PINK1 using an anti-PINK1 antibody. (**C**) YFP-Parkin expressing HeLa cells were treated with or without 1μM O/A for 1 and 2 hours. Subsequently, the cells were harvested for immunoprecipitation with anti-PINK1 or OGT antibody and analyzed by western blotting. (**D**) HEK293T cells were transfected with OGT-FLAG and/or PINK1-MYC as indicated for 48 h and were lysed with IP lysis buffer. The cell lysates were subjected to MYC IP or FLAG IP and analyzed by western blotting. (**E**) HEK293T cells were pretreated with OSMI-1 for 24 h and treated with 1 μM O/A for 2h. WGA was used to pull down O-GlcNAcylated proteins, and then immunoblot to detect using the indicated antibody. (**F**) PINK1 is O-GlcNAcylated *in vitro*. GST-PINK1 was incubated overnight with GST-tagged OGT and 5 mM UDP-GlcNAc, then immunoblotted with the antibodies.

Given that O-GlcNAcylation correlates with the protein level of PINK1, we hypothesized that PINK1 is subjected to O-GlcNAcylation by OGT. To test this hypothesis, we first performed wheat germ agglutinin (WGA) pull-down followed by GlcNAc elution of whole-cell extracts from YFP-HeLa and HEK293T cells. WGA binds to terminal GlcNAc residues, and we found an enhanced level of PINK1 pull-down by WGA in response to O/A treatment in YFP-HeLa and HEK293T cells (Figure 5A-5B), indicating an increased level of PINK1 O-GlcNAcylation. We also performed a pull-down assay using an anti-O-GlcNAc antibody in HEK293T and YFP-HeLa cells and found an increased level of O-GlcNAcylated PINK1 (Figure S5). Next, we examined the protein-protein interaction by performing an endogenous IP assay and demonstrated that PINK1 mutually interacted with OGT in cells treated with O/A (Figure 5C). Reciprocal co-IP assays showed the same results (Figure 5D). Furthermore, inhibition of OGT by treatment with OSMI-1 markedly reduced the O-GlcNAcylation level of PINK1 as well as LONP1 in YFP-HeLa cells (Figure 5E). Similar results were found for VDAC, another OMM protein that has been reported to be O-GlcNAcylated 38. To further demonstrate whether PINK1 is subject to O-GlcNAcylation by OGT, we conducted *in vitro* GlcNAcylation assay by incubating recombinant GST-PINK1 with GST-tagged OGT and UDP-GlcNAc, and results from this assay further confirmed O-GlcNAcylation of PINK1 by OGT (Figure 5F).

### O-GlcNAcylation of PINK1 on Ser229 promotes PINK-Parkin-mediated mitophagy

**Figure 6:**
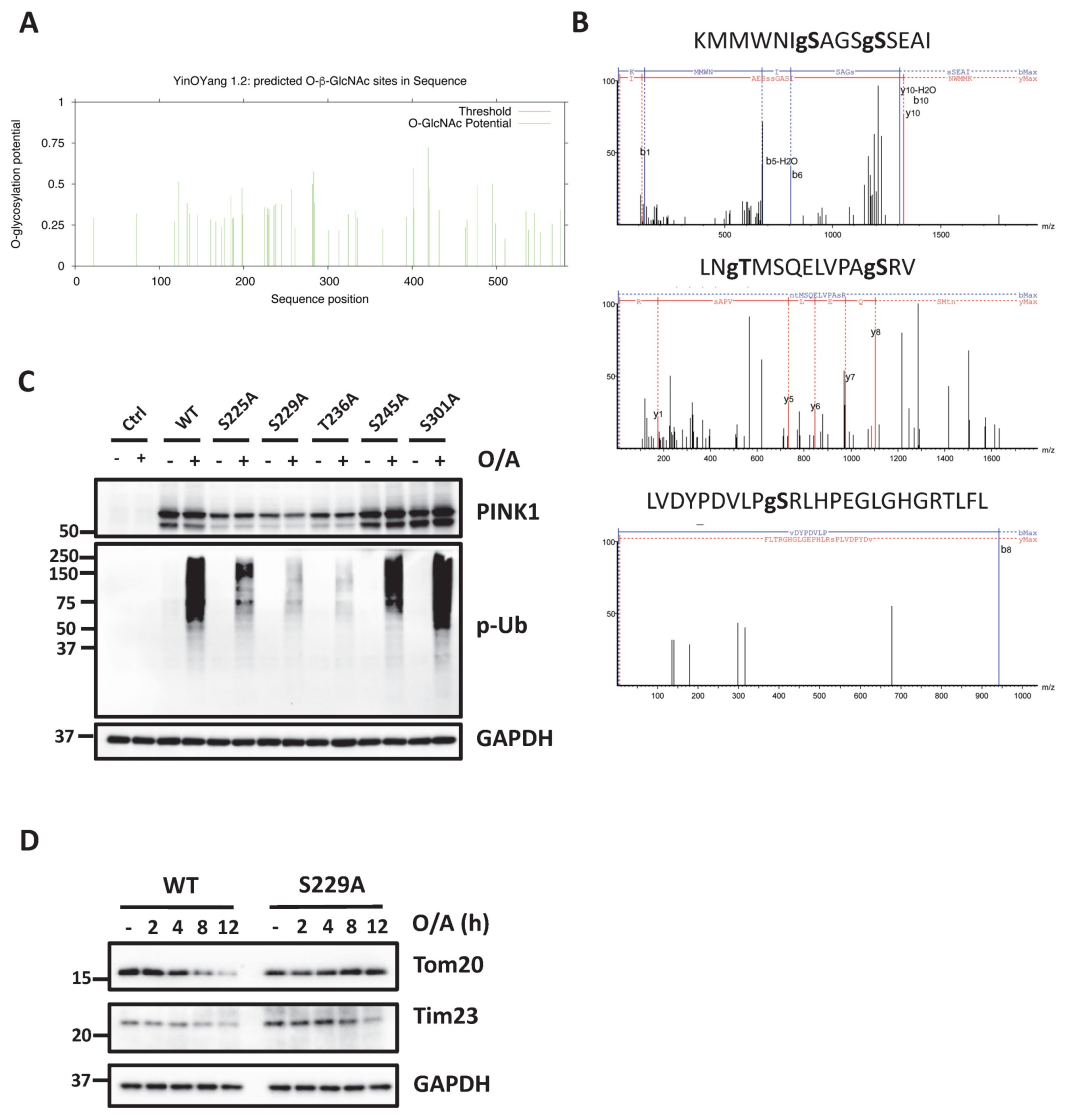
** PINK1 Serine 229 mediated PINK1-parkin-mediated mitophagy.** (**A**) Graphics of PINK1 O-GlcNAcylation using computational methods based on the development of PTM-specific databases (https://services.healthtech.dtu.dk/services/YinOYang-1.2/). (**B**) Site mapping of the PINK1 O-GlcNAcylation modification site in peptides KMMWNISAGSSSEAI, LNTMSQELVPASRV, LVDYPDVLPSRLHPEGLGHGRTLFL with LC-MS/MS analysis. (**C**) PINK1 WT, S225A, S229A, T236A, and S301A mutants were expressed in HeLa PINK1-/- stably expressing mCherry-Parkin cells following O/A treatment for 2 hours, subjected to western blot with PINK1 and phospho-ubiquitin. (**D**) PINK1 WT and S229A were transfected in HeLa PINK1-/- stably expressing mCherry-Parkin cells, the time course for mitochondrial proteins detection upon treatment with O/A. Scale bar=10μm.

After demonstrating the increased level of O-GlcNAcylation of PINK1, here we aimed to identify the specific O-GlcNAcylation sites of PINK1. First, we performed O-GlcNAc database analysis with the YinOYang server 39 for predictions of O-GlcNAc attachment sites and found 52 potential O-GlcNAc modification sites of PINK1 (Figure 6A). Using this O-GlcNAc analysis procedure, over 50 O-GlcNAcylation sites in PINK1 were mapped to PINK1 S/T residues (Figure S6). To further determine the sites of PINK1 O-GlcNAcylation, O-GlcNAcylated PINK1 was analyzed by mass spectrometry following immunoprecipitation and enrichment of this protein. Three peptides KMMWNISAGSSSEAI (amino acids 219-233), LNTMSQELVPASRV (amino acids 234-247), LVDYPDVLPSRLHPEGLGHGRTLFL (amino acids 292-316) were identified as carrying O-GlcNAc moieties (Figure 6B). In this segment, five amino acids (Ser225, Ser 229, Thr236, Ser245, and Ser 301) could be the putative O-GlcNAc sites for PINK1. To validate the above findings, we created PINK1 with serine/threonine to alanine mutation at these sites and tested the effects of those mutants by reconstitution in PINK1-KO HeLa cells. Under the O/A-induced mitophagy condition, reconstitution with the S225A, S229A and S236A mutant significantly reduced PINK1 and p-Ub compared to the WT PINK1 (Figure 6C). Since S229A mutant exhibits the most striking decrease in PINK1 and p-Ub upon O/A treatment, we assessed whether the degradation of mitochondrial proteins such as Tom20 and Tim23 was suppressed in PINK1 KO cells expressing S229A mutant. As expected, O/A-induced degradation of Tom20, Tim23 was significantly inhibited in cells with overexpression of S229A mutant (Figure 6D). Consistently, reconstitution of PINK1 with S229A mutant effectively blocked O/A-induced Parkin recruitment to damaged mitochondria (Figure S7A, B). We did not observe significant differences in MMP in cells with reconstitution of WT and S229A mutant PINK1 (Figure S7C, D). Furthermore, S225A mutation reduced the PINK1 and p-Ub level and suppressed O/A-induced mitochondrial degradation (Figure S7E). Taken together, data from this part of our study clearly demonstrate that PINK1 undergoes O-GlcNAcylation as an important mechanism in the control of mitophagy.

### Glucose metabolism promotes mitophagy via O-GlcNAcylation

**Figure 7:**
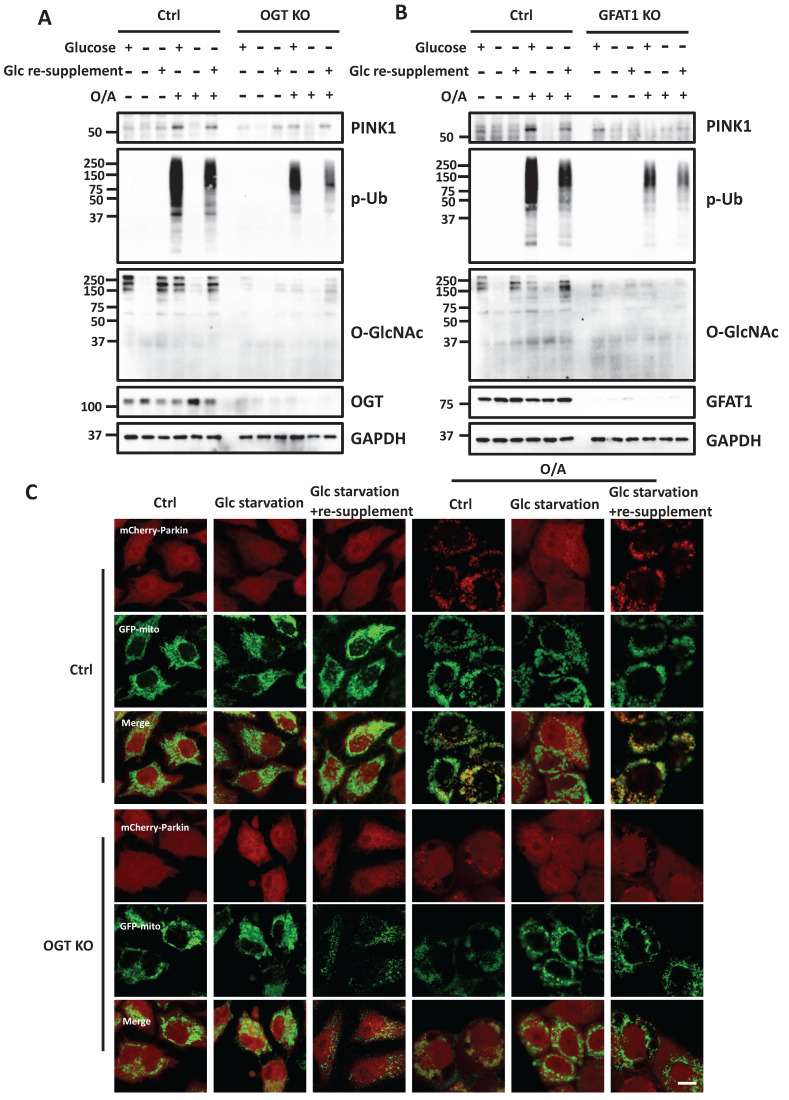

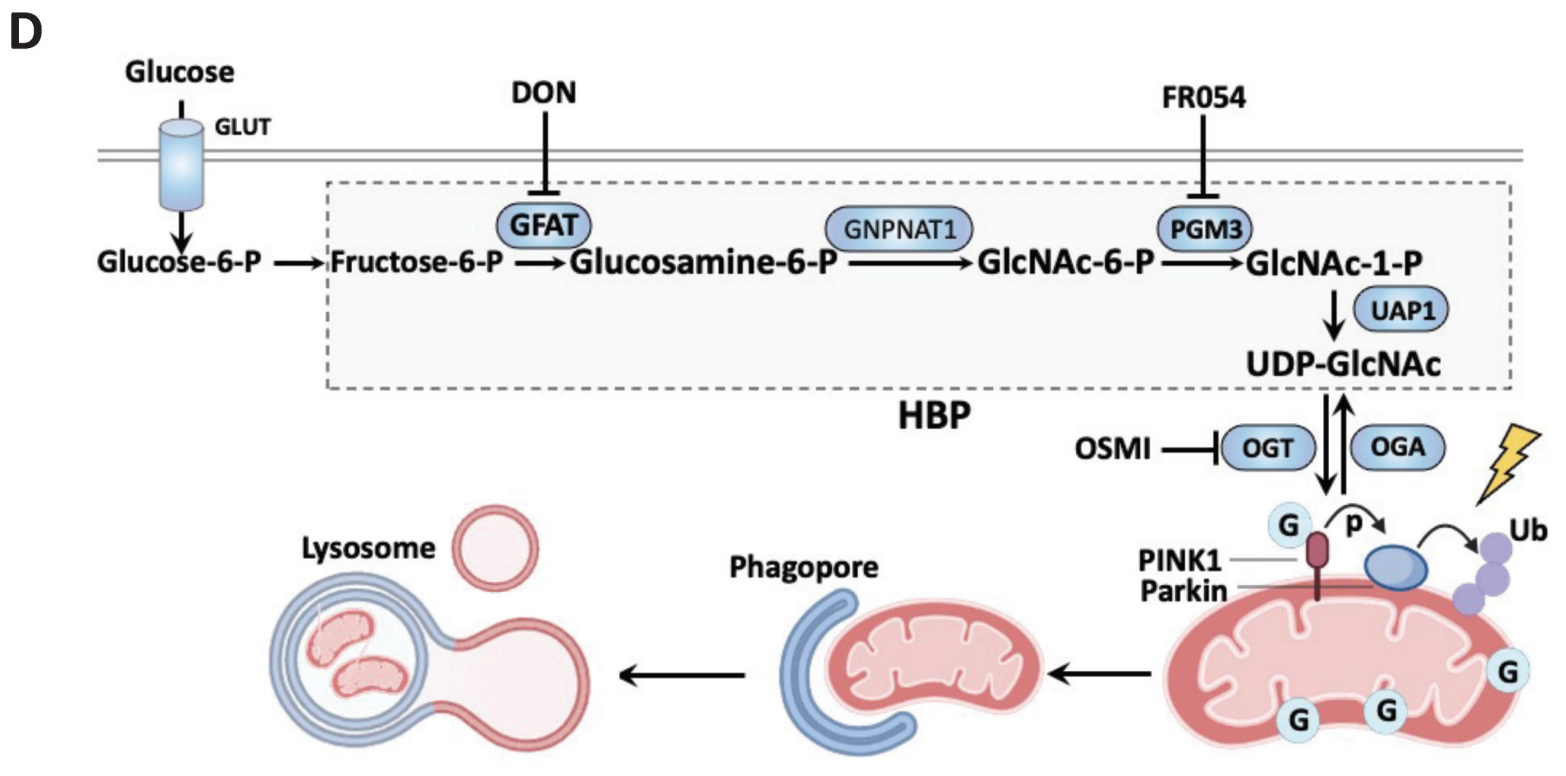
** Glucose regulates mitophagy via protein O-GlcNAcylation.** (**A**) The control and OGT KO HeLa stably expressing mCherry-Parkin were treated with glucose-containing or glucose-free DMEM for eight hours, and the starved cells were further incubated with glucose for 8 h. Then the cells were stimulated with O/A for 2 h before collecting. PINK1, p-Ub and O-GlcNAcylation expression levels under the above treatment display a significant response to glucose starvation. (**B**) Similar treatments were examined in GFAT1 KO cells upon O/A-induced mitophagy. (**C**) Parkin mitochondrial translocation and GFP-mitochondrial were monitored by confocal microscopy in control and OGT KO HeLa cells stably expressing mCherry-Parkin and GFP-mitochondrial. Scale bar=10μm. (**D**) Schematic model for the positive regulatory role of O-GlcNAcylation in mitophagy**.** Glucose is fluxed through the HBP via a series of metabolic enzymes, including GFAT, GNPNAT1, PGM3 and UAP1, producing UDP-GlcNAc for O-GlcNAcylation. O-GlcNAcylation is a crucial positive modulator of mitophagy that promotes PINK1 stability and its activation to mediate PINK1/Parkin-dependent mitophagy. Importantly, glucose metabolism is an important regulator of mitophagy, mainly via O-GlcNAcylation in response to mitochondrial damage. The schematic model is created with BioRender.com.

After establishing the role of O-GlcNAcylation in mitophagy, we hypothesized that HBP and O-GlcNAcylation are implicated as the underlying mechanisms in glucose-dependent mitophagy. To test this hypothesis, we performed glucose starvation and supplement assays to test the role of O-GlcNAcylation in mitophagy. Consistent with an earlier report 29, we found that glucose starvation completely prevented full-length PINK1 stabilization and abolished p-Ub levels induced by O/A. Re-supplement of glucose to glucose-deprived medium effectively restored both full-length PINK1 and p-Ub (Figure 7A). Interestingly, in OGT KO cells, the rescue effect of glucose re-supplementation on PINK1 and p-Ub levels was significantly impaired, suggesting that the positive regulatory effect of glucose on mitophagy is mainly via OGT-mediated O-GlcNAcylation. Importantly, similar effects were found in GFAT1 KO cells (Figure 7B). In OGT KO cells, we further examined the pattern of Parkin recruitment to mitochondria and found that glucose re-supplement failed to rescue Parkin recruitment following O/A treatment (Figure 7C and S7F). Collectively, data from this part of our study indicate that O-GlcNAcylation mediated by OGT and GFAT1 is a key underlying mechanism for the positive regulatory function of glucose metabolism in mitophagy.

## Discussion

In this study, we have demonstrated a novel mechanism linking glucose metabolism with PINK1-Parkin-dependent mitophagy via O-GlcNAcylation. First, we found that acute mitochondrial damage promotes glucose uptake and increases the overall protein O-GlcNAcylation level. Next, inhibition of O-GlcNAcylation by pharmacological or genetic approaches targeting OGT and OGA leads to the suppression of mitophagy. Mechanistically, we provided evidence that PINK1 is a substrate of O-GlcNAcylation in mitophagy. We further identified the sites of PINK1 O-GlcNAcylation, and mutation of these sites effectively abolished the effect of GlcNAcylation on PINK1 and mitophagy. More importantly, we found that O-GlcNAcylation is one of the key mechanisms underlying the regulatory role of glucose metabolism in mitophagy. Taken together, results from this study reveal a novel regulatory mechanism in mitophagy via O-GlcNAcylation of PINK1, which provides a new link between glucose metabolism and mitophagy, as summarized in Figure 7D.

Autophagy is a dynamically regulated and canonical nutrient-sensing regulatory mechanism in cellular physiology, during which O-GlcNAc modifications are involved in multiple phases of autophagy machinery—including ULK1 O-GlcNAcylation functions in the initiation of autophagy, and direction of the O-GlcNAcylated SNAP29 to autophagosome maturation 40-42. Mitophagy is a selective form of autophagy for the clearance of dysfunctional mitochondria via the autophagy-lysosome pathway 43. At present, the exact function of OGT-mediated O-GlcNAcylation in mitophagy remains obscure. On one hand, there is evidence showing the negative role of GlcNAcylation in mitophagy. For instance, O-GlcNAcylation functions as a negative regulator in control of mitochondrial fragmentation and mitophagy by directly modifying ferritin in response to ferroptosis 28. On the other hand, there is convincing evidence demonstrating the positive role of O-GlcNAcylation in mitophagy. OGT is critical for HSC maintenance by facilitating the accumulation of H3K4me3 at the Pink1 transcription start site, although PINK1 did not rescue downstream progenies and mature hematopoietic cells in OGT-deficient cells 27. Although the experiments were mostly performed in U2OS cells responding to ferroptotic stress and the model cells did not express detectable Parkin 44, these studies have pointed to a causative link between O-GlcNAcylation and PINK1-dependent mitophagy. Our data are generally consistent with a recent study that OGT plays a positive role in mitophagy via promoting the expression of PINK1 and LC3 and O-GlcNAcylation of mitochondrial proteins 45.

PINK1 is the most important factor in the initiation of mitophagy following acute mitochondrial damage induced by inhibitors of the ETC such as A/O and the uncoupler CCCP 46. The molecular mechanisms underlying PINK1 activation have been extensively studied, and several important PTMs have been reported, including phosphorylation, ubiquitination, and oxidation 47-49. Once the full-length PINK1 is stabilized at OMM, it forms a homodimer and undergoes autophosphorylation, an important process for the full activation of PINK1 47, 50. There is a major ubiquitination site in PINK1 (lysine 137), and this form of PTM is also implicated in its kinase activity and mitophagy 48. In addition, under oxidative stress conditions, PINK1 also undergoes Cys oxidation at the ATP-binding pocket of PINK1, promoting its kinase activity 47. Based on the well-known fact, phosphorylation at Ser228 and Ser402 residues within the kinase domain of PINK1 is the primary mechanism for kinase activity 49, 51. Data from our study revealed another form of PTM, the O-GlcNAcylation of PINK1 in the course of mitophagy. We not only identified the sites of O-GlcNAcylation, but also demonstrated the functional relevance of this PTM in determining PINK1's stability and the outcome of mitophagy. Thus, our study adds a new level of complexity in the regulation of PINK1 during mitophagy. Then the key question lies in whether the kinase activity and protein stability of PINK1 are regulated by O-GlcNAcylation. OGT-mediated O-GlcNAcylation is critical for protein stability, although the detailed mechanisms remain unclear 52, 53. For example, knockdown of OGT specifically decreased the protein stability of EZH2, partly through the downregulated binding OGT to the substrate 52. OGT has reported roles in tight enzyme-substrate interactions, unlike other short-term hit-and-run interactions. It is not surprising to speculate PINK is unstable, since the binding of OGT and PINK1 is destroyed when OGT is inhibited. It is reasonable to hypothesize that O-GlcNAcylation regulates the phosphorylation or ubiquitination of PINK1, promoting protein stability. Moreover, we will need to consider that O-GlcNAcylation of PINK1 promotes the kinase activity via rendering the oxidation less active. Taken together, one possibility is that the O-GlcNAc-defective PINK1 was simply vulnerable to degradation due to the spatially limited manner. Another possibility is that is that the O-GlcNAcylation of PINK1 alters the other PTMs, contributing to PINK1 stability or kinase activity. At present, there is evidence suggesting the presence of extensive cross-talk between O-GlcNAcylation and phosphorylation 20, 25, 54. The exact details that explain how PINK1 stability is regulated and accesses the kinase activity are currently under investigation.

It is interesting that O-GlcNAcylation contributes to PINK1 stabilization at mitochondria, either genetic intervention of OGT or O-GlcNAc mutant of PINK1 is proved to suppress PINK1 level. In canonical mitophagy, PINK1 accumulates on the mitochondria and recruits Parkin to the mitochondrial surface to initiate mitophagy 3. The genetic deletion and pharmacological inhibition of OGT had moderate effects on Parkin translocation to the mitochondria, but strong effects on PINK1 and p-Ub. The above finding suggests a potential impairment of mitophagy that is independent of Parkin when OGT is inhibited. There are many mechanisms by which cells remove their dysregulated mitochondria to maintain mitochondrial quality control 55. It can be grouped into three main types including mitophagy-dependent, mitophagy-independent pathways and secretion of mitochondria 3. Recently, the mitochondria lysosome-related organelle (MLRO) is a non-canonical mechanism for mitochondrial quality control 56, 57. It is beneficial to consider the alternative mechanism in which OGT-mediated PINK1-dependent mitophagy could be involved.

Glucose metabolism is one of the essential metabolic processes that affect a variety of cellular functions. As mentioned earlier, glucose metabolism has been implicated in the regulation of mitophagy. Apparently, glucose metabolism has multiple ways to regulate the mitophagy process. First, glucose metabolism is known to regulate mitophagy by maintaining intracellular ATP levels and PINK1 expression 29. PINK1 can also undergo cleaved and subsequent degradation when glucose is sufficient, since mitochondrial is in normal condition. PINK1 stabilization requires ATP from active glucose metabolism in response to mitochondria depolarization such as O/A treatment. Second, in diabetic mice, increased levels of PINK1 and Parkin were found in all layers of the vascular wall under sustained hyperglycemia, leading to the higher level of mitophagy 58. Third, key enzymes in governing glucose metabolism such as HK2, and PKM2, play a positive role in regulating mitophagy 30, 31. Moreover, one of the ongoing works in our laboratory has identified Glucose-6-phosphate dehydrogenase (G6PD), a rate-limiting enzyme in the pentose phosphate pathway (PPP), an integral part of glycolysis, as an important regulator of mitophagy. In addition to the importance of glucose metabolism in mitophagy, glucose uptake and the following protein O-GlcNAcylation are promoted acute upon mitochondrial damage. MG132 treatment in the presence of O/A predominantly increased O-GlcNAcylation of mitochondrial proteins while decreasing cytosolic proteins compared with O/A treatment. Apart from a small amount of proteins encoded by the mitochondrial genome, most proteins residing in the mitochondria are nuclear-encoded and synthesized in the cytosol 59. Proteasome inhibitor MG132 caused mitochondrial oxidative stress and fragmentation 60. It is reasonable that some O-GlcNAcylated proteins involved in mitochondrial stress are synthesized in the cytosol. These O-GlcNAcylated proteins are not necessary for protein function in the cytosol but are essential for mitochondrial function under stress and fragmentation. These proteins with cytosolic O-GlcNAcylation could translocate to the mitochondria in this process. In this study, we expand the scope of functional interactions between glucose metabolism and mitophagy by establishing the role of O-GlcNAcylation (Figure 7D).

Understanding the role of O-GlcNAcylation in mitophagy opens up a new window for the development of novel interventional strategies in the modulation of mitophagy and of new therapeutic approaches in mitophagy-related diseases such as neurodegeneration and cancer.

## Methods

### Cell lines

Experiments were performed in HEK293T and HeLa from the American Type Culture Collection (ATCC). HeLa cells stably expressing YFP-Parkin (YFP-HeLa) and YFP-HeLa cells stably expressing mito-Keima were kind gifts from Dr. Richard Youle. HeLa cells stably expressing mCherry-Parkin and GFP-mitochondrial were established in our lab 61. Cells were cultured in Dulbecco's modified Eagle's medium (DMEM) with 4.5 g/L glucose supplemented with 10% fetal bovine serum (FBS) and 1% penicillin/streptomycin. All cell lines were incubated at 37°C with 5% CO_2_.

### Reagents and antibodies

Reagents used in this research: Oligomycin (Merck, 11,342), Antimycin A (Merck, A8674), 2-NBDG (Thermo Fisher Scientific, N13195), MG132 (Med Chem Express, HY-13259), bafilomycin A1 (BafA1; Merck, B1793), carbonyl cyanide 3-chlorophenylhydrazone (CCCP; Merck, C2759), OSMI-1-1 (Med Chem Express, HY-119738), Bortezomib (Selleckchem, S1013), Proteinase K (Med Chem Express, HY-108717), Triton X-100 (Merck, X100), PMSF (Beyotime, ST506), Anti-c-Myc Magnetic Beads (Med Chem Express, HY-K0206), Anti-Flag Magnetic Beads (Med Chem Express, HY-K0207), Wheat Germ Agglutinin (WGA) Agarose(Vector laboratories, AL1023s), PEI (Polysciences, 24885-2), Immobilon ECL Ultra Western HRP Substrate (Millipore, WBULS0500), 4% Paraformaldehyde Fix Solution (Solarbio, P1110), Recombinant P. humanus PINK1 Protein (R&D systems, AP-182), Recombinant human OGT Protein R&D systems, 8446-GT), UDP-GlcNAc (Merck, U4375), 6-Diazo-5-oxo-L-nor-Leucine (DON) (Med Chem Express, HY-108357), FR054 (Med Chem Express, HY-124909A), JC-1 (Beyotime, C2006).

Primaries antibodies used in this research: anti-CTD110.6 (Cell signaling technology, 9875s), anti-Ubiquitin (Cell signaling technology, 43124), anti-GFAT1 (Cell signaling technology, 5322), anti-OGT (Cell signaling technology, 24083), anti-Tom20 (Cell signaling technology, 42406s), anti-PINK1 (Cell signaling technology, 6946), anti-Phospho-Ubiquitin (Ser65) (Cell signaling technology, 62802), anti-Myc (Cell signaling technology, 2276), anti-DYKDDDDK (Cell signaling technology, 14793), anti-LONP1 (Cell signaling technology, 28020), anti-VDAC (Cell signaling technology, 4661), anti-LC3B (Cell signaling technology, 3868), anti-p62 (Cell signaling technology, 39749), anti-MFN1 (Cell signaling technology, 14739), anti-HSP60 (Cell signaling technology, 12165), anti-OGA (Abcam, ab124807), anti-GAPDH (Proteintech, 60004-1-Ig), anti-Tom40 (Proteintech, 18409-1-AP), anti-Tim23 (BD Biosciences, 611223), anti-CISD1 (Proteintech, 16006-1-AP), anti-MT-CO2 (Abcam, ab110258), anti-PGM3 (Invitrogen, PA5-56250), anti-O-GlcNAc (RL2) [AlexaFluor 647] (Novus Biologicals, NB300-524AF647).

Secondary antibodies used in this research: Peroxidase-conjugated affinity pure goat anti-mouse IgG, light chain specific (Jackson Immuno Research, 115-035-174), peroxidase-conjugated IgG fraction monoclo- nal mouse antirabbit, light chain specific (Jackson Immuno Research, 211-032-171), Alexa Fluor-594 goat anti-rabbit IgG (H+L) Highly Cross-Adsorbed Secondary Antibody (Thermo fisher scientific, Cat# A-11037).

### Mitochondria fractionation and topology analysis

Mitochondria were purified by sequential centrifugation as reported previously. Topology analysis of OGT and GFAT1 was formed by proteinase K protection assay. Subsequently after isolation, the mitochondrial pellet was resuspended in MIM buffer (280 mM sucrose with 10 mM HEPES, pH 7.2). Purified mitochondria were dissolved in MIM buffer with or without digitonin (0.1%) and concentration of proteinase K (5 μg/ml) for 15 min on ice. Samples without proteinase K or with 1% Triton X-100 were provided as controls. The reaction was stopped by the addition of 10 mM PMSF and samples were boiled with 4x Laemmli loading buffer.

### Generation of knockdown cells and knockout cell lines with CRISPR/Cas9

To generate YFP-HeLa and HEK293T cells stably knockdown OGT, shRNA targeting OGT sequence is CCGGTTTAGCACTCTGGCAATTAAACTCGAGTTTAATTGCCAGAGTGCTAAATTTTTG, AATTCAAAAATTTAGCACTCTGGCAATTAAACTCGAGTTTAATTGCCAGAGTGCTAAA. Lentiviral particles were generated by transfecting HEK293T cells with pLKO.1 puro-OGT shRNA construct, psPAX2 and pMD2.G (Addgene) at a ratio of 7:5:2. Viral supernatants were collected 48h following transfection and pellets were removed using the centrifuge at 2,000g for 5min. YFP-Parkin-expressing HeLa and HEK293T cells were transduced with lentivirus for 48h. Cells were then selected with 2μg/ml puromycin for one week. To generate knockout lines in mCherry-Parkin/mitoGFP expressing cells, cells transduced to stably express Cas9 using the lentiviral system (pFU-Cas9-T2a-miRFP670) were established in our lab. Cells were subsequently transfected with Alt-R® CRISPR-Cas9 sgRNA containing guide sequences against the gene of interest. SgRNA targeting OGT are #1 CCGTGCACCACTGCGTGATT, #2 CATCGATGGTTATATTAACC, #3 GTTGGCACATCGAGAATATC. SgRNA targeting GFAT1 is TTGGAATAGCTCATACCCGT. SgRNA targeting OGA is GGTGTGGATAGCAACGTAGT. Cells were verified for knockout efficiency by western blotting.

### Immunoblotting and immunoprecipitation assays

The harvested cells were lysed in SDS lysis buffer (62.5 mM Tris-HCl, pH 6.8, 25% glycerol, 2% SDS, 1× phosphatase and proteinase inhibitor cocktail. Lysates were diluted in 4 x Laemmli sample buffer and boiled for 5 min. Equal amounts of proteins were subjected to 10% or 12% SDS-PAGE gels, followed by transfer to PVDF membranes. Membranes were blocked with superblock for one hour, incubated overnight with primary antibodies, and then incubated with secondary antibodies. Bands were then visualized by chemiluminescence with the Bio-rad ChemiDoc System.

HEK293T cells were transfected with the indicated plasmids. After 48h, cells were lysed with IP lysis buffer. Lysates were then centrifuged at 13000 × rpm for 10 min. For the myc and flag immunoprecipitation, the supernatant was incubated with myc/flag-magnetic beads for 4h. For other conditions, the 25μl protein A/G magnetic beads were incubated with antibodies following the instructions of antibody datasheets overnight with gentle rotation at 4°C and lysates were subsequently incubated with protein A/G coupled to the corresponding antibody for an additional 4 h. For pulling down O-GlcNAcylated proteins, cell lysates were incubated with 20μl succinylated wheat germ agglutinin (sWGA)-conjugated beads at 4°C for four hours. The immunoprecipitates were then combined with 2x Laemmli sample buffer by boiling at 100 °C for 5 min. The samples were resolved on gels and analyzed by immunoblotting.

### Intracellular staining of O-GlcNAc

The analysis of intracellular O-GlcNAc by flow cytometry was performed originally as described in Murakami et al. (Murakami et al, 2021), briefly, cells were treated with O/A or CCCP for the indicated time prior to being lifted off the 24-well plates. Cells were collected by centrifugation at 500 x g for 5 min. Further, cells were fixed with 4.0% paraformaldehyde (PFA) for 10 min and subjected to permeabilization with 0.5% saponin (Macklin). Then cells were harvested in the dark and incubated in AlexaFluor 647-conjugated anti-O-GlcNAc antibody at the concentration of 3 μg/μl in 100 μl of 0.5% Saponin containing dilution buffer for 30 min. Cell analysis was performed by flow cytometry.

### Cytosolic and mitochondrial fractionation

Cytosolic and mitochondrial fractionation was adapted from Guang L et al., 62 as follows: YFP-HeLa cells were lysed in 500μl Digitonin buffer (150mM NaCl, 50mM HEPES pH7.4, 25μg/ml Digitonin, Protease and phosphatase inhibitors) and gently agitated on a rotator at 4°C for 10 min. The homogenates were centrifuged at 2,000xg for 10 min at 4°C. Supernatants were obtained and further centrifuged sequentially three times at 20,000xg for 20 min at 4°C, transferring supernatants to new pre-chilled tubes following each centrifugation to finally obtain cytosolic proteins. The cytosolic fraction was ready for immunoblot analysis. The pellet from the initial centrifuge was resuspended in ice-cold PBS to wash away the remaining Digitonin buffer. The suspension was centrifuged at 2,000xg for 5 min at 4°C. The supernatant was discarded and pellets were resuspended in 100μl NP-40 buffer (150mM NaCl, 50mM HEPES pH7.4, 1% NP-40, protease and phosphatase inhibitors) and mixed on ice for 30 min. Samples were centrifuged at 7,000xg for 10 min at 4°C to yield the crude mitochondria fraction for immunoblot analysis. The cytosol and mitochondrial fraction were quantified from the cells for normalization.

### Immunofluorescence microscopy

Cells seeded in 35 mm plates were treated as indicated in Figure legends. After treatment, cells were rinsed in PBS and fixed with 4% paraformaldehyde at room temperature (RT) for 10 min. Cells were washed with PBS twice. For immunostaining, samples were permeabilized with 0.1% Triton X-100 in PBS for 10 min at room temperature and blocked with 1% BSA in PBST(PBS+ 0.1% Tween 20) for 1 h at RT. Then, cells were incubated with 1% BSA in PBST supplemented with antibody (HSP60, 1:300) overnight at 4°C. Cells were then washed with PBS 3 times and incubated with Alexa 594-conjugated secondary antibodies for 1 h at RT. For cells expressing fluorescent tagged proteins, cells were seeded as above. After treatments, cells were fixed and washed 3 times with PBS before image visualization. Images were visualized using 63x oil immersion lens on an LSM 710 and 880 Airyscan microscope (Zeiss). For mitochondrial recruitment of Parkin analysis, Fiji JACoP plugin was adopted to analyze the Mander's overlap coefficient values as described previously 63.

### Glucose uptake assay

For fluorometric measurement of glucose uptake *in vitro* assay, YFP-HeLa and HeLa cells were washed with PBS for 3 times firstly, and cultured in glucose free DMEM with the indicated treatments. Later, the cells were incubated with 200μM 2-NBDG in glucose-free media for 30 min at 37°C. Wash thrice by centrifugation at 500 x g at 4°C for 5 min. The cells were resuspended in 300 μl PBS in the dark and subjected to flow cytometry. Finally, the average fluorescence intensity (MFI) 2-NBDG signal was further analyzed using CytoFLEX S Flow Cytometer (BD).

### *In vitro* O-GlcNAc assay

*In vitro* O-GlcNAcylation of PINK1 was performed in 20 ml assay volumes containing 2μg OGT, 1μg PINK1 in reaction buffer (50mM Tris-HCl, pH 7.5, 1mM DTT, 12.5mM MgCl_2_), and 1mM UDP- GlcNAc. The reactions were incubated for overnight at 37°C, stopped by adding 4x SDS-PAGE sample buffer, subjected to SDS-PAGE, transferred to PVDF membrane and then detected with indicated antibodies.

### Mass spectrometry

HEK293T cells at 70% confluence in a 10-cm dish were transfected with 14 μg PINK1-myc plasmid containing C-myc-tagged PINK1, and cultured for 48 h with overnight 10 μM Thiamet G and O/A for 2 h. PINK1-myc was purified by immunoprecipitation as described above, and resolved by SDS-PAGE and stained with Coomassie blue staining. Cut three excised gel slices from each gel into 1 mm^3^ cubes and transfer the gel cubes to a 1.5-mL microcentrifuge tube. The samples were processed by Biotech-Pack company. The digested protein was subjected to Nano LC-MS/MS analysis on Nanoflow UPLC: Easy-nLC1200 system with Orbitrap Eclipse^TM^ Mass Spectrometer (Thermo Fisher Scientific, USA). The raw MS files were analyzed and searched against the target protein database based on the species of the samples using PEAKS Studio 10.6. The parameters were set as follows: the protein modifications was HexNAcylation (S,T), the enzyme specificity was set to chymotrypsin or trypsin; the maximum missed cleavages were set to 3; the precursor ion mass tolerance was set to 20 ppm, and MS/MS tolerance was 0.02 Da. Only high confident identified peptides were chosen for downstream protein identification analysis.

### JC-1 and TMRE staining

The indicated cells were stained with JC-1 (1x) or TMRE (20nM) for 30 min in working solution at 37°C. Cells were collected and washed twice in PBS, then analyzed by flow cytometry under both FITC and PE channels. For JC-1 assays to assess mitochondrial membrane potential, we measured the JC-1 monomers and aggregates. For TMRE fluorescence intensity, relative levels of mitochondrial membrane potential were determined.

### Statistics analysis

The statistical significance of the differences observed more than three samples were performed by One-way ANOVA with Dunnett's multiple comparisons test using GraphPad Prism 9 software. Two-way ANOVA with Sidak's multiple comparisons test was used to compare multiple groups of two factors. Error bars are expressed as mean ± standard deviation. Data are presented as means of the results of at least 3 replicated experiments. Statistical significances of P < 0.05 were considered significant.

## Supplementary Material

Supplementary figures.

## Funding

Grants to H.M. Shen from CPG2023-0032-FHS, CPG2024-0035-FHS, UM-MYRG2020-00022-FHS, and from Macau Science and Technology Development Fund (FDCT0078/2020/A2, FDCT0031/2021/A1, FDCT0081/2022/AMJ, FDCT 0004/2021/AKP, FDCT0036/2024/RIB1); Grants to Z.W. Xu from National Natural Science Foundation of China (grant no. 32000903), and the Scientific Research Foundation of Jiangsu University for Senior Professional Talents (20JDG48).
