# Supplementary material for: Coupling of glucose metabolism with mitophagy via O-GlcNAcylation of PINK1
Source: Int J Biol Sci. 2025 Jun 20;21(9):4252–69. doi: 10.7150/ijbs.112672 (PMC12224001; doi:10.7150/ijbs.112672)
Supplement: Supplementary file 1 — Supplementary figures. [file ijbsv21p4252s1.pdf]

Supporting Information

Coupling of glucose metabolism with mitophagy via O-GlcNAcylation of PINK1

Zhiwei Xu Xiangzheng Gao, Dade Rong, Jingyao Wang, Liangliang Gao, Mingzhu Tang, Yiguan Chen, Yichi Zhang, Liming Xie, Liming Wang, Guang Lu, Jia-Hong Lu, Wei Liu, Han-Ming Shen

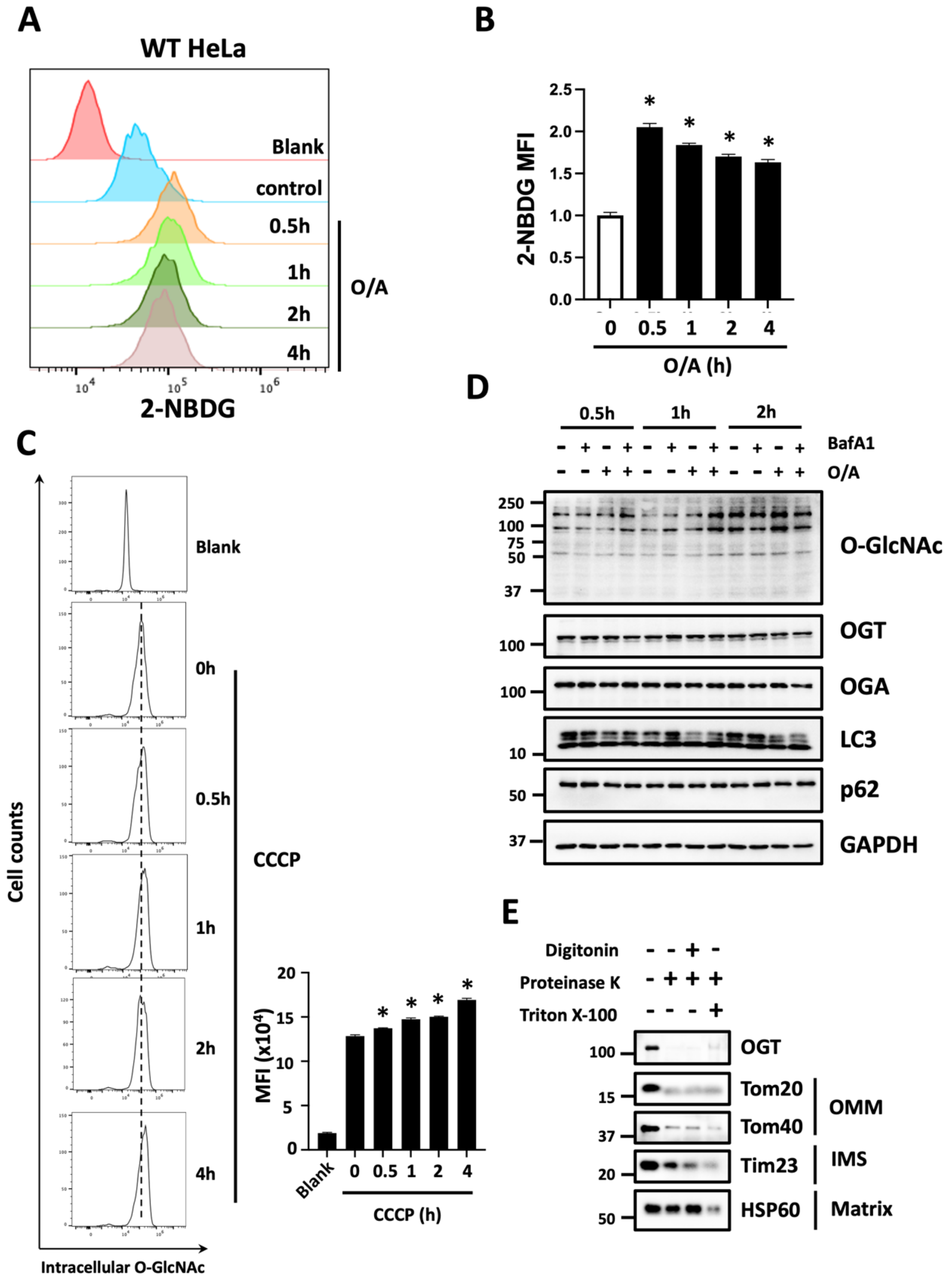

Figure S1. Increased glucose uptake and O-GlcNAcylation in WT HeLa cells following O/A-induced mitophagy. (**A and B**) Flow cytometry assay of glucose uptake by WT HeLa cells in the 2-NBDG assay. (**C**) Flow cytometric analysis of intracellular O-GlcNAc levels in YFP-HeLa treated with CCCP (20 μM) for 0.5 to 4 h. (**D**) YFP-HeLa cells were pretreated with or without Bafilomycin A1 (BafA1,100nM) for 1 h. Subsequently, the cells were treated with or without O/A (1μM and 1μM) for 0.5, 1, 2h

and subjected to western blotting analysis with the indicated antibodies. (**E**) Topology assay showing OGT localization at the OMM. Purified mitochondria were isolated from YFP-Parkin-HeLa cells and treated with the indicated reagents of proteinase K and digitonin. *$p < 0.05$.

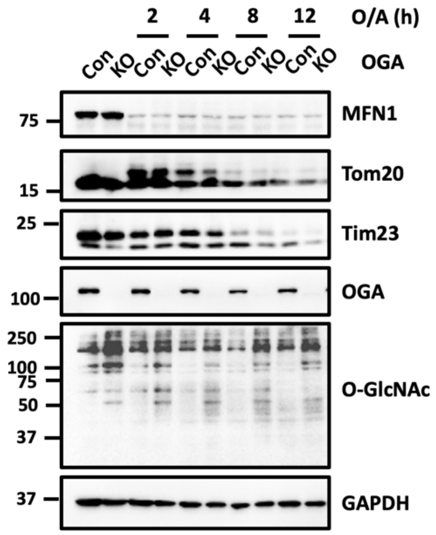

Figure S2. OGA KO did not regulate mitophagy. Mcherry-Parkin expressing control and OGA KO cells HeLa cells treated with 1 μM O/A, then harvested at the indicated time points for western blotting analysis with the indicated antibodies.

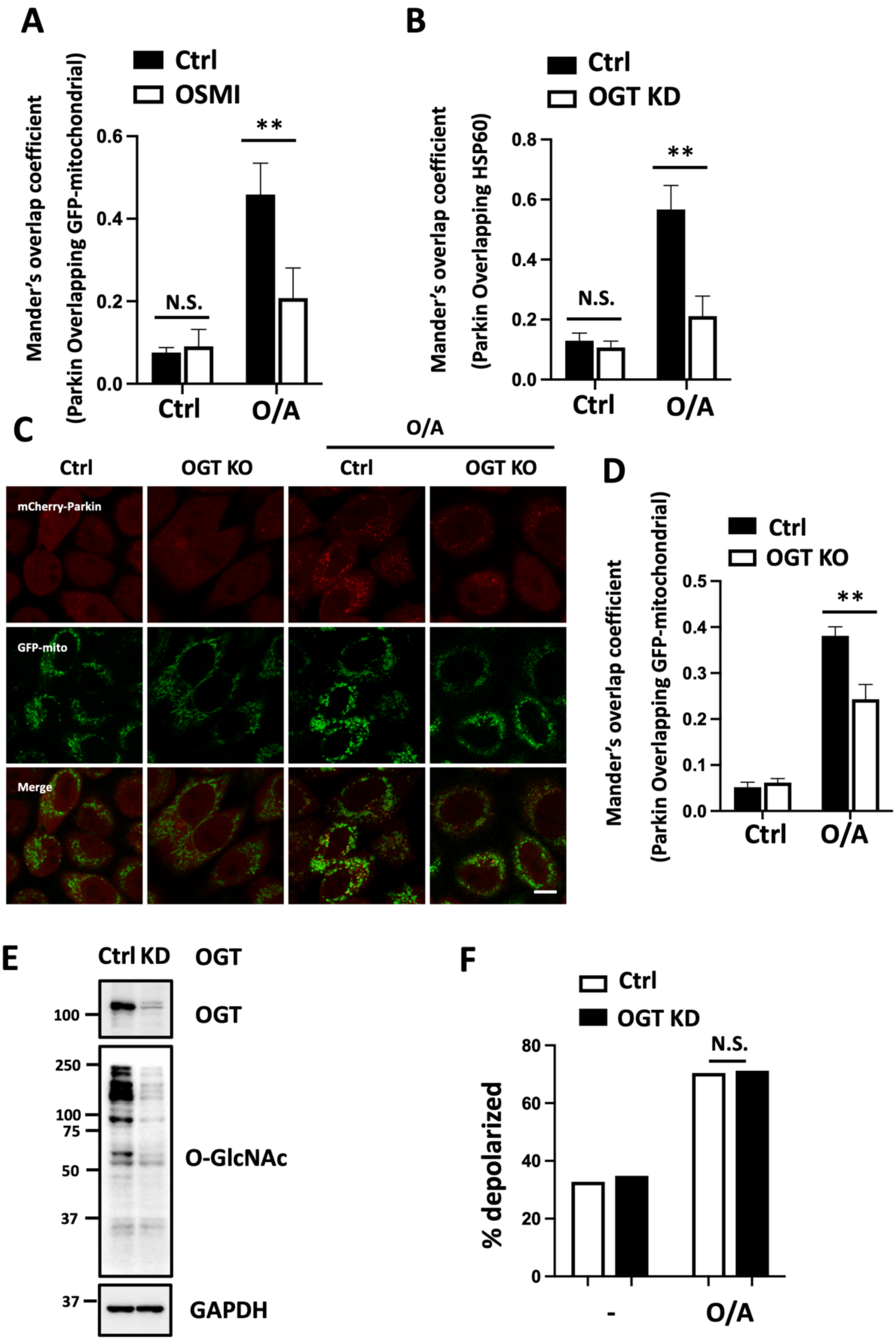

Figure S3. OGT KO reduced Parkin mitochondrial translocation. (**A and B**) Colocalizations between mCherry-Parkin and GFP-mitochondrial, YFP-Parkin and mitochondrial marker HSP60 as in (Fig 3D and 3F) were analyzed by Mander's overlap coefficient. (**C**) Control and OGT-KO HeLa stably expressing mCherry-Parkin upon treatment with or without O/A for 2h. Representative immunofluorescence images of WT and OGT-KO cells. Scale bar=10μm. (**D**) Colocalization between mCherry-Parkin

and GFP-mitochondrial as in (Fig S3C). (**E**) Control or OGT KD cells were lysed for western blotting analysis to confirm the knockdown effects of OGT. (**F**) JC-1 staining and quantification of Ctrl and OGT KD HEK293T treated with 1 μM O/A or DMSO for 6h. **, $p < 0.01$, N.S., no significance.

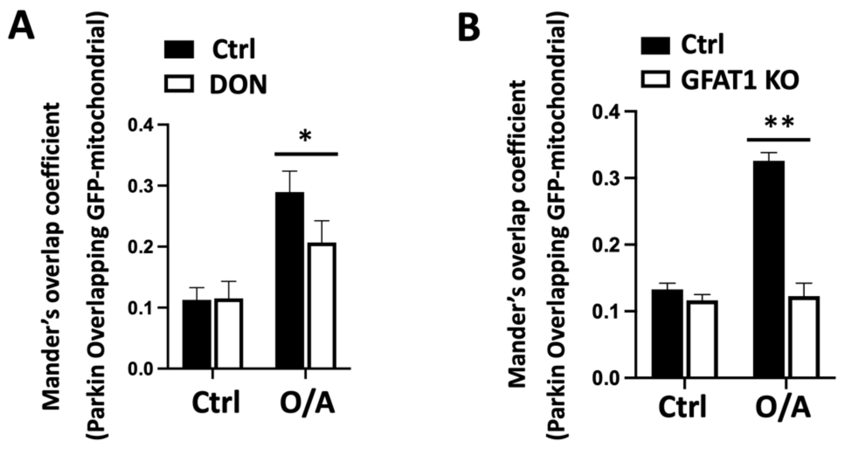

Figure S4. Colocalization between mcherry-Parkin and GFP-mitochondrial as in (Fig 4B and 4D) was analyzed by Mander's overlap coefficient. (N.S., no significance; *, $P < 0.1$, **, $P < 0.01$; Two-way ANOVA with Sidak's multiple comparisons test).

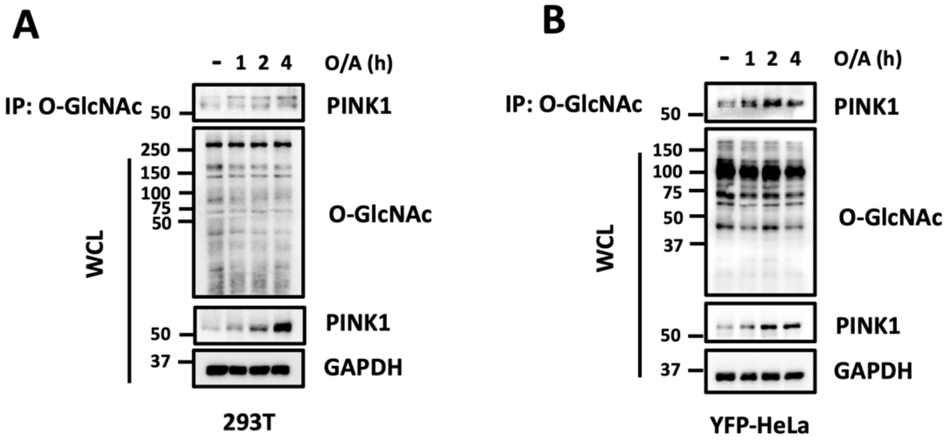

Figure S5. PINK1 undergoes O-GlcNAcylation. (**A and B**) HEK293T and YFP-HeLa cells following O/A for the indicated time were subjected to immunoprecipitation with anti-O-GlcNAc antibody. Then the immunoprecipitation was blotted with PINK1 antibody and lysates were subjected to O-GlcNAc antibody respectively.

SeqName	Residue	O-GlcNAc result	Potential (o-glcnac)
NP_115785.1	22 T	+++	0.2932
NP_115785.1	73 S	+++	0.3181
NP_115785.1	118 S	+++	0.2733
NP_115785.1	123 S	+++	0.5152
NP_115785.1	133 T	+++	0.3832
NP_115785.1	136 S	+++	0.3192
NP_115785.1	145 T	+++	0.3103
NP_115785.1	161 S	+++	0.2817
NP_115785.1	167 S	+++	0.2708
NP_115785.1	174 T	+++	0.2371
NP_115785.1	177 T	+++	0.2923
NP_115785.1	185 T	+++	0.4256
NP_115785.1	187 S	+++	0.2909
NP_115785.1	188 T	+++	0.2929
NP_115785.1	198 T	+++	0.4757
NP_115785.1	199 S	+++	0.3143
NP_115785.1	225 S	+++	0.3586
NP_115785.1	228 S	+++	0.3546
NP_115785.1	229 S	+++	0.3112
NP_115785.1	230 S	+++	0.3480
NP_115785.1	236 T	+++	0.3678
NP_115785.1	238 S	+++	0.3788
NP_115785.1	245 S	+++	0.3743
NP_115785.1	257 T	+++	0.4696
NP_115785.1	261 S	+++	0.2329
NP_115785.1	282 T	+++	0.5008
NP_115785.1	283 S	+++	0.5747
NP_115785.1	284 S	+++	0.3839
NP_115785.1	301 S	+++	0.2168
NP_115785.1	313 T	+++	0.2176
NP_115785.1	324 T	+++	0.3166
NP_115785.1	333 T	+++	0.3340
NP_115785.1	335 S	+++	0.2933
NP_115785.1	365 S	+++	0.2254
NP_115785.1	393 S	+++	0.2999
NP_115785.1	401 S	+++	0.5954
NP_115785.1	402 S	+++	0.3498
NP_115785.1	419 S	+++	0.7224
NP_115785.1	420 T	+++	0.4713
NP_115785.1	432 S	+++	0.3413
NP_115785.1	463 S	+++	0.2361
NP_115785.1	465 S	+++	0.2813
NP_115785.1	477 S	+++	0.4912
NP_115785.1	495 S	+++	0.5014
NP_115785.1	499 S	+++	0.2598
NP_115785.1	510 S	+++	0.1658
NP_115785.1	535 S	+++	0.3341
NP_115785.1	538 T	+++	0.2207
NP_115785.1	545 T	+++	0.3223
NP_115785.1	552 T	+++	0.2053
NP_115785.1	566 T	+++	0.2546
NP_115785.1	576 S	+++	0.3408

Figure S6. The predicted sites of PINK1 could be modified by O-GlcNAcylation. Computational O-GlcNAcylation sites of PINK1 predicted via YinOYang prediction program. This prediction program characterizes PINK1 O-GlcNAcylation over 50 different sites, where its prediction strengths are marked with high [“+++”], ranging from low to high [“+” to “++++”] in the program. The prediction strength depends on the sequence condition and conformation.

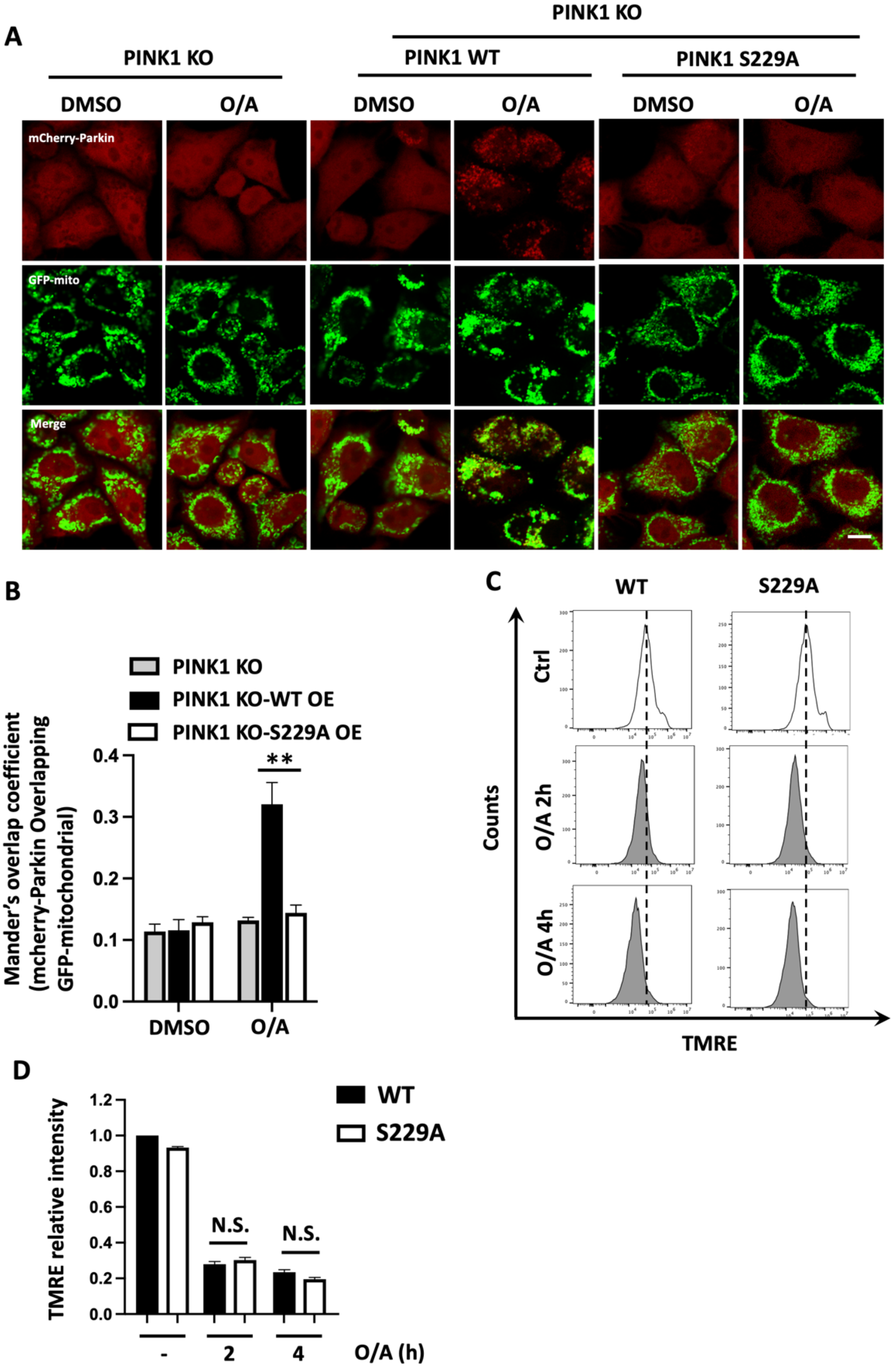
A
PINK1 KO
PINK1 KO
PINK1 WT
PINK1 S229A
DMSO
O/A
DMSO
O/A
DMSO
O/A
mCherry-Parkin
GFP-mito
Merge
B
PINK1 KO
PINK1 KO-WT OE
PINK1 KO-S229A OE
Mander's overlap coefficient (mcherry-Parkin Overlapping GFP-mitochondrial)
0.4
0.3
0.2
0.1
0.0
**
DMSO
O/A
C
WT
S229A
Ctrl
O/A 2h
O/A 4h
Counts
TMRE
D
WT
S229A
TMRE relative intensity
1.2
1.0
0.8
0.6
0.4
0.2
0.0
N.S.
N.S.
-
2
4
O/A (h)

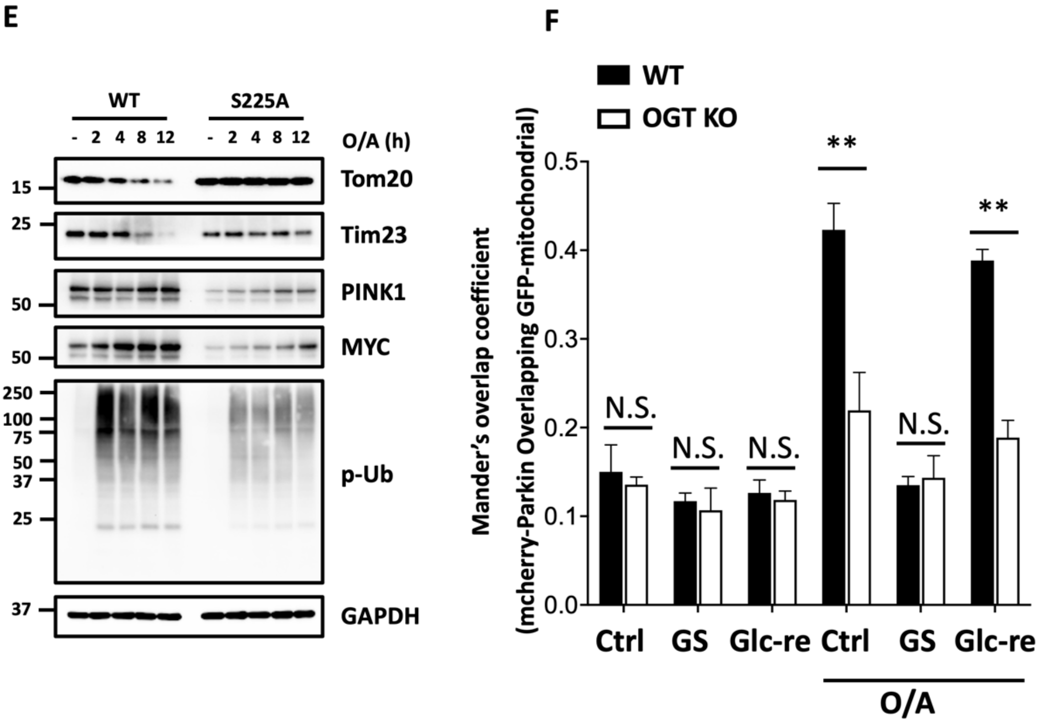

Figure S7. S229A and S225A mutant suppress PINK1-Parkin-mediated mitophagy. (**A**) PINK1 KO cells transfected with WT PINK1 or S229A mutant were treated with or without O/A for 2h. (**B**) Colocalization between mCherry-Parkin and GFP-mitochondrial as in (Fig S7A) was analyzed. (**C and D**) TMRE staining and quantification of WT PINK1 and S229A mutant overexpressing YFP-HeLa PINK1 KO cells treated with 1 μM O/A or DMSO for 2 and 4 h. (**E**) PINK1 WT and S225A were transfected in HeLa PINK1−/− stably expressing mCherry-Parkin cells, time course for mitochondrial proteins detection upon treatment with O/A. $*p < 0.05$. (**F**) Colocalization between mcherry-Parkin and GFP-mitochondrial as in (Fig 7C) was analyzed by Mander's overlap coefficient. (N.S., no significance; **, $P < 0.01$; Two-way ANOVA with Sidak's multiple comparisons test. GS, glucose starvation, Glc-re, glucose re-supplement). Scale bar=10μm.
